# Supplementary material for: Insights into the Influence of Signal Peptide on the Enzymatic Properties of Alginate Lyase AlyI1 with Removal Effect on Pseudomonas aeruginosa Biofilm
Source: Mar Drugs. 2022 Nov 29;20(12):753. doi: 10.3390/md20120753 (PMC9787728; doi:10.3390/md20120753)
Supplement: Supplementary file 1 [file marinedrugs-20-00753-s001.zip › marinedrugs-2004676-supplementary.pdf]

marine drugs

MDPI

Article

Insights into the Influence of Signal Peptide on the Enzymatic Properties of Alginate Lyase AlyI1 with Removal Effect on *Pseudomonas aeruginosa* Biofilm

Ming-Jing Zhang [1], Shuai-Ting Yun [1], Xiao-Chen Wang [1], Li-Yang Peng [1], Chuan Dou [2] and Yan-Xia Zhou *

[1] Marine College, Shandong University, Weihai 264209, China
[2] Shangdong Kelun Pharmaceutical Co., Ltd, Bingzhou 256600, China
* Correspondence: zhouyx@sdu.edu.cn

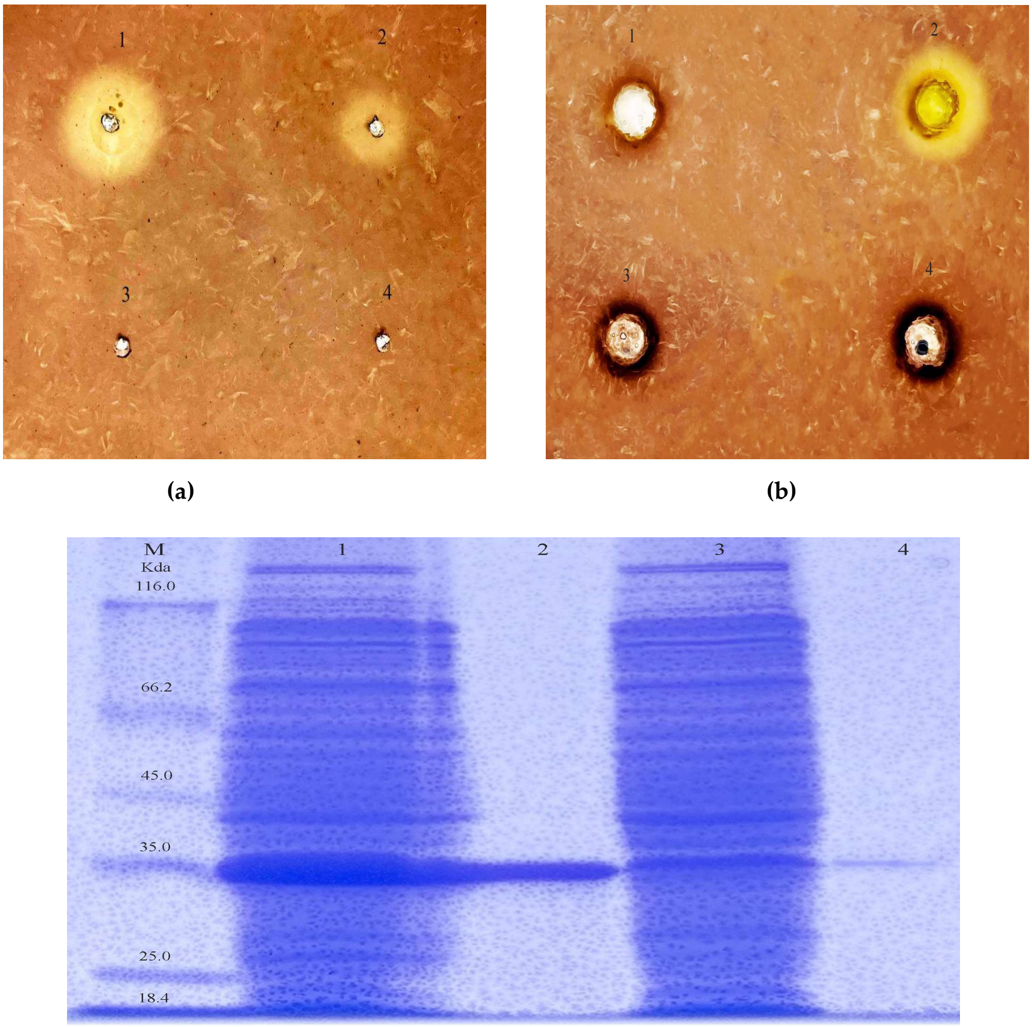

(c)

Figure S1. Expression, purification of rALYI1/rALYI1-1. (a) Detection of alginate lyase activity of rALYI1. (1) the purified protein, (2) the induced group protein, (3) Empty vector control, (4) noninduced control (b) Detection of alginate lyase activity of rALYI1-1. (1) the purified protein, (2) the induced group protein, (3) Empty vector control, (4) noninduced control (c) SDS-PAGE analysis of the recombinant rALYI1/rALYI1-1 expression and purification. Lane M, protein marker; Lane 1, recombinant protein rALYI1-1; Lane 2, recombinant protein rALYI1; Lane 3, the purified rALYI1.
